# Acceleration of the excitation decay in Photosystem I immobilized on glass surface

**DOI:** 10.1007/s11120-017-0454-z

**Published:** 2017-10-13

**Authors:** Sebastian Szewczyk, Wojciech Giera, Rafał Białek, Gotard Burdziński, Krzysztof Gibasiewicz

**Affiliations:** Department of Physics, Adam Mickiewicz University, ul. Umultowska 85, Poznan, 61-614 Poland

**Keywords:** Photosystem I, Transient absorption spectroscopy, Excitation energy transfer, Cyanobacteria, Conductive glass, Biophotovoltaics, Red chlorophylls

## Abstract

Femtosecond transient absorption was used to study excitation decay in monomeric and trimeric cyanobacterial Photosystem I (PSI) being prepared in three states: (1) in aqueous solution, (2) deposited and dried on glass surface (either conducting or non-conducting), and (3) deposited on glass (conducting) surface but being in contact with aqueous solvent. The main goal of this contribution was to determine the reason of the acceleration of the excitation decay in dried PSI deposited on the conducting surface relative to PSI in solution observed previously using time-resolved fluorescence (Szewczyk et al., Photysnth Res 132(2):111–126, 2017). We formulated two alternative working hypotheses: (1) the acceleration results from electron injection from PSI to the conducting surface; (2) the acceleration is caused by dehydration and/or crowding of PSI proteins deposited on the glass substrate. Excitation dynamics of PSI in all three types of samples can be described by three main components of subpicosecond, 3–5, and 20–26 ps lifetimes of different relative contributions in solution than in PSI-substrate systems. The presence of similar kinetic components for all the samples indicates intactness of PSI proteins after their deposition onto the substrates. The kinetic traces for all systems with PSI deposited on substrates are almost identical and they decay significantly faster than the kinetic traces of PSI in solution. We conclude that the accelerated excitation decay in PSI-substrate systems is caused mostly by dense packing of proteins.

**Electronic supplementary material:**

The online version of this article (doi:10.1007/s11120-017-0454-z) contains supplementary material, which is available to authorized users.

## Introduction

In recent years, photosynthetic pigment–protein complexes gained great attention in fabrication of bio-inorganic devices. Therefore, deep insight into the nature and behavior of these complexes in different environments (e.g., immobilized onto various substrates) is crucial for their utilization. Among all photosynthetic particles, Photosystem I (PSI) is one of the most commonly used photosensitive materials in miscellaneous prototype bio-devices, due to its overall high stability and high photon-to-electron conversion quantum yield near unity (Gobets and van Grondelle 2001). In nature, it catalyzes the trans-membrane electron transfer upon irradiation, and thus it can be considered as a natural optoelectronic device in nanoscale. The structure and properties of this complex are well documented in the literature (Fromme and Grotjohan 2008; Caffarri et al. 2014; Nelson and Junge 2015). Among 12 protein subunits and 127 cofactors per cyanobacterial PSI monomer, which include 96 chlorophylls *a* (Chls *a*), 22 carotenoids (Cars), two phylloquinones, three iron–sulfur clusters, four lipids, one Ca^2+^ metal ion, and 201 water molecules, few of them compose the reaction center (RC) where charge separation process occurs (Jordan et al. 2001). The RC cofactors are the following: 6 Chls (two of them forming P700—primary donor, two accessory Chls labeled A, and two primary electron acceptors labeled A_0_), two phylloquinones, and three terminal iron–sulfur cofactors (Fx, Fa, Fb). The role of the remaining pigments is to form an antenna system which collects the light energy. Although primary donor absorption maximum is at 700 nm, a great majority of Chls absorb at around 680 nm (“bulk” Chls). In various PSI complexes, a small pool of pigments that absorb at wavelengths longer than 700 nm is present (“red” Chls). Their nature, origin, and presumptive location in *Synechocystis* were extensively studied in the literature and briefly discussed previously (Szewczyk et al. 2017). A recent, detailed review on red Chls in different cyanobacteria can be found in Karapetyan et al. (2014).

The usefulness of PSI complex in various bio-hybrid devices has been previously demonstrated in many studies. These include different substrates (for working electrodes) such as gold, TiO_2_, ZnO, graphene, and conductive polymer systems (for instance, Ciesielski et al. 2010; Mershin et al. 2012; Feifel et al. 2015; Carter et al. 2016; Robinson et al. 2017). In these contributions, it was proposed that electron flow direction in devices based on high-carrier concentration electrodes (gold, modified graphene, FTO—fluorine-doped tin oxide, or ITO—indium-doped tin oxide) is determined mostly by orientation of the PSI near the electrode, due to the unidirectional electron transfer in PSI—from P700 to Fb. Theoretically, in the case of disordered assembly of the PSI monolayer onto surface, the observed net photocurrent density can be decomposed into two major, opposing contributions, which correspond to cathodic (electron injected from the working electrode substrate to P700^+^) or anodic (electron injected from Fb^−^ to the substrate) photocurrent. In the aforementioned studies, different net photocurrent, under various experimental conditions, was observed, which may be related to the degree of asymmetry of cathodic and anodic photocurrents. However, there may be other factors limiting the overall photocurrent related to electrical contact between proteins and substrate, charge recombination reactions, and, last but not least, very basic energy and electron transfer properties of PSI immobilized on the substrate.

In our previous paper, we reported that immobilization of PSI on inorganic substrate, FTO conductive glass (a plate of glass covered with a thin, optically transparent, conductive layer of FTO), modifies the spectral characteristics of some chlorophyll pools and accelerates the decay of PSI antenna excitation: from mean lifetime of t_av_ = 16 ps for PSI in solution to 11 ps for PSI deposited on FTO glass (Szewczyk et al. 2017). A possible reason was proposed to be an additional quenching process resulting from electron injection from PSI to the conductive FTO substrate. It is well established that excited states of dyes being in contact with semiconducting surfaces are quenched very efficiently due to electron transfer between the dye molecule and the substrate (for instance, Tachibana et al. 1996; Grätzel et al. 2005; Sobuś et al. 2015; Ashford et al. 2015). The electron injection rates for dyes immobilized on semiconducting surfaces like TiO_2_, ZnO, ZrO_2_, or Al_2_O_3_ are often extracted from measurements of dye-excited states’ lifetimes (Wenger et al. 2005; Koops et al. 2009, Sobuś et al. 2014, 2015; Idigoras et al. 2015). Still, however, the nature and timescale of electron injection from dyes most commonly used in dye-sensitized solar cells photovoltaics, ruthenium or indoleum, are a matter of debate. Some contributions considered biphasic injection kinetics—the faster component with lifetime < 100 fs, and the slower one occurring on picosecond to nanosecond timescale (Koops et al. 2009; Idigoras et al. 2015; Sobuś et al. 2015), related with electron transfer from the singlet and triplet state of the dye, respectively. Other studies proposed that slower component is a result of a transfer from loosely attached, more distant, or aggregated molecules (Wenger et al. 2005). The exact values of electron injection rates depend on the surface type and experimental conditions. However, acceleration of the dye-excited state quenching was generally correlated with more efficient electron injection into a particular substrate (Koops et al. 2009; Sobuś et al. 2014).

Another considered cause of accelerated excitation dynamics in immobilized PSI (Szewczyk et al. 2017) was radical disparity in environmental conditions (PSI in water + detergent solution vs. PSI dialyzed in order to get rid of detergent and then deposited onto semiconductor and dried). Due to drying and formation of a densely packed multilayer PSI film during the deposition process, new interactions between complexes may arise which change their spectroscopic (and energetic) properties. Red chlorophylls are examples of natural states that appear as a result of strong interaction between pigments, leading to a mixing of excitonic and charge transfer states (Romero et al. 2008; Novoderezhkin et al. 2016). An artificial formation of three extra red states in densely packed PSI monomers that are deposited on FTO glass and dried was demonstrated (Szewczyk et al. 2017). To our knowledge, there is no literature describing the influence of dehydration on the first steps of energy transfer in PSI; however, it was shown that water density near electron transfer cofactors may affect the protein flexibility, charge screening, and P700^+^ reduction rate (Dashdorj et al. 2005). A possibility of altering hydrogen bonds and, therefore, changes in structure which may lead to aggregation was discussed in the aforementioned contribution, but interestingly the FTIR analysis of severely dehydrated sample excluded changes in secondary structure of PSI (Sacksteder et al. 2005). In another contribution (Malferrari et al. 2016), it was demonstrated that the relative humidity indeed modulates electron transfer to iron–sulfur clusters. Despite this, the dried PSI complexes remained stable over a long period. In the case of purple bacteria, it was shown that the decay rate of the excited bacteriochlorophyll dimer (P*) was slightly increased due to drastic drying of the purple bacterial RC film (Yakovlev et al. 2012).

**Fig. 1:**
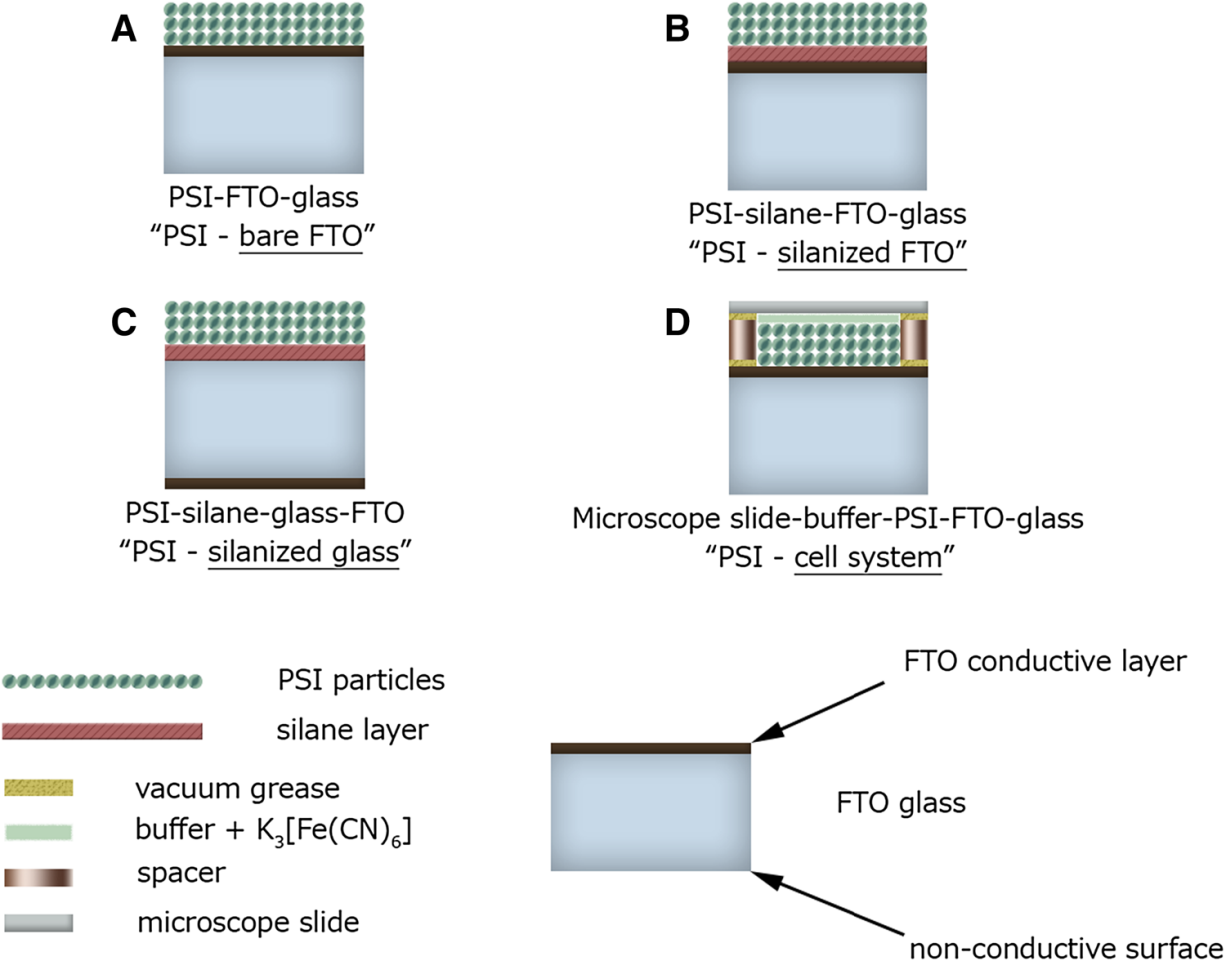
Illustration of the PSI-immobilized systems used in the study. **a** PSI deposited on the conductive surface of the FTO glass. **b** PSI deposited on the silane-covered conductive surface of the FTO glass. **c** PSI deposited on the silane-covered non-conductive surface of the FTO glass. **d** PSI deposited on the conductive surface of the FTO glass in the cell-like system with aqueous electrolyte

In order to reveal the factors responsible for the observed accelerated de-excitation in immobilized PSI and taking into account the above considerations, we decided to investigate excitation dynamics in PSI, using femtosecond transient absorption in 3-ns time window, under four different environmental conditions: (1) PSI suspended in aqueous solution; (2) PSI immobilized and dried on FTO conductive glass (Fig. 1a); (3) PSI immobilized and dried on non-conducting surface of silanized FTO glass (Fig. 1b); and (4) PSI immobilized on FTO conductive glass and being in contact with aqueous solution (photovoltaic cell-like system with a thin layer of aqueous solution between PSI-covered FTO glass and a coverglass; Fig. 1d). The experiments were performed for both trimeric and monomeric forms of PSI from *Synechocystis* sp. PCC 6803.

Femtosecond transient absorption measurements for wild-type PSI from *Synechocystis* in aqueous solution have been performed previously (Hastings et al. 1994; Melkozernov et al. 2000; Savikhin et al. 2000; Shelaev et al. 2010). In general, the excitation dynamics in PSI core complex can be described using three major components: (1) subpicosecond, which describes excitation equilibration, (2) 2–6 ps related to both photochemical quenching of bulk chlorophylls and equilibration between bulk and red chlorophylls, and (3) 21–26 ps ascribed to excitation trapping in reaction center (photochemical quenching of excitation equilibrated over bulk and red chlorophylls). Experiments described in the aforementioned studies were performed under excitation in the red region (predominantly 660 nm), for trimeric PSI form, in a range of excitation pulse energies.

## Materials and methods

### Isolation of PSI particles

The wild-type *Synechocystis* sp. PCC 6803 strain was used to obtain monomeric and trimeric forms of PSI. All chemicals used were purchased from Sigma-Aldrich (reagent or analytical grade). Cells were grown at 25 °C in BG-11 liquid medium (Rippka et al. 1979), in ambient air, under stirring and continuous white light. Thylakoids were obtained by sonication, preceded by incubation with lysozyme. PSI particles were extracted using* n*-dodecyl-*β*-d-maltoside (*β*-DM) and purified by ion-exchange chromatography, as described earlier in detail (Szewczyk et al. 2017). Isolated PSI complexes were resuspended in buffer A containing 20 mM Bis–Tris (pH 7.0), 5 mM MgCl_2_, 5 mM CaCl_2_, 10 mM NaCl, and 0.03% β-DM (v/v) and stored at − 20 °C until use. The obtained samples were characterized using fluorescence correlation spectroscopy (FCS) and 77 K steady-state fluorescence techniques. The former revealed high homogeneity of fractions (explicit differences in diffusion coefficients for trimers and monomers), and the latter revealed high purity of PSI complexes’ emission peak for both monomers and trimers at 720 nm with no significant fluorescence from PSII at wavelengths shorter than 695 nm.

### Immobilization of the PSI particles onto substrates

**Fig. 2:**
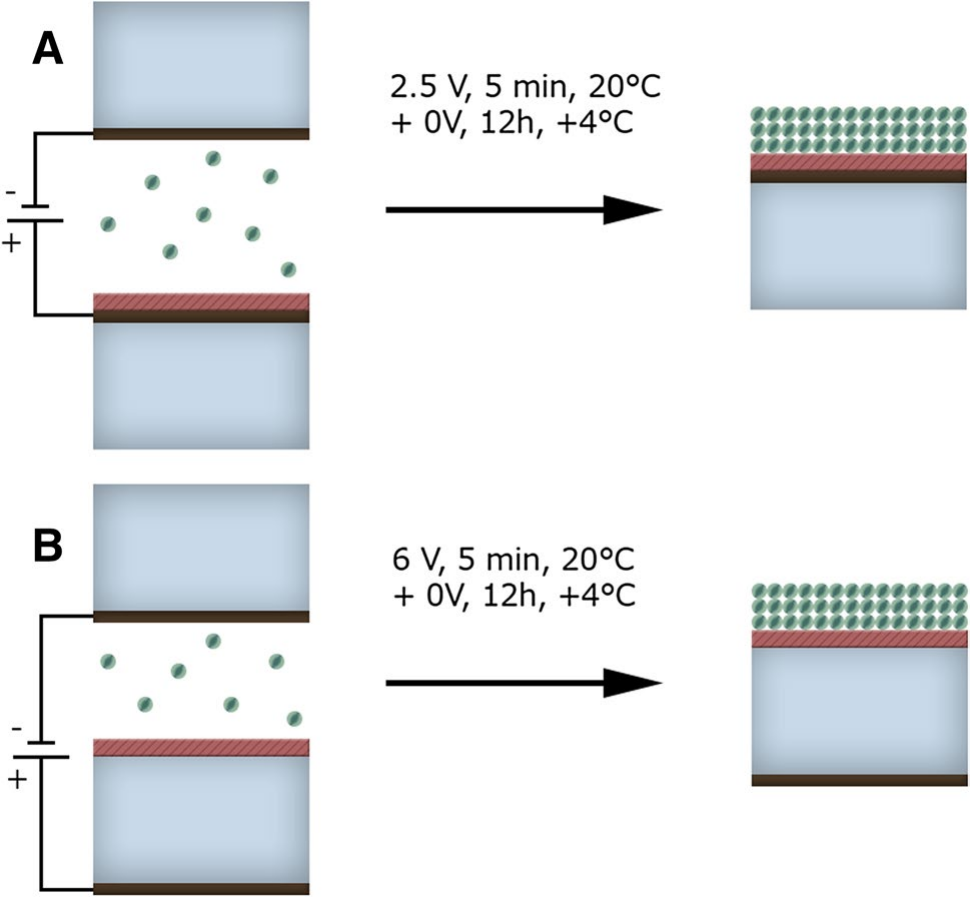
Procedure of deposition of PSI complexes onto conductive (**a**) and non-conductive (**b**) surfaces of FTO glass covered with a silane layer

The deposition of the PSI particles onto FTO conductive glass (Fig. 1a) was performed as described previously (Szewczyk et al. 2017). To briefly summarize this process, PSI complexes were washed by ultracentrifugation and dialyzed in order to get rid of β-DM and salts. Next, a small volume of sample (40 µl of PSI solution, OD_680 nm,_ 1 cm ~ 1.5) was placed between two FTO glass electrodes and the immobilization process was assisted by electric field (2.5 V, 5 min; Fig. 2a). After drying at 4 °C for ~ 12 h, a transparent and durable film of area of ~ 0.25 cm^2^ was obtained. The samples were stored at 4 °C until measurements. The estimated number of PSI layers deposited onto FTO was 10–30, assuming the cross-section size of a PSI monomer of the order of 100 nm^2^. The thickness of the FTO glass was 2.2 mm and the thickness of the FTO conductive layer was 200 nm.

In some experiments, the conductive surface of FTO glass substrate was covered with silanes which formed a layer of insulator eliminating the possibility of electron injection from PSI into the FTO substrate (Fig. 1b). In another set of control experiments, the non-conductive surface of glass substrate was covered with silanes in order to increase its hydrophobicity (Figs. 1c, 2b). We discovered that high hydrophobicity of the substrate is crucial for successful deposition of an optically homogenous film of PSI complexes. The conductive surface of FTO glass is hydrophobic enough in order to obtain a homogenous film of PSI without silanization.

Before silanization, the glass slides were washed carefully by sonication in ethanol for 10 min and then annealed at 100 °C for 30 min. Silanization of substrates was performed using two procedures: (1) chemical vapor deposition (CVD, Moo-Yeal 2016) and (2) spin-coating (Moo-Yeal 2016). CVD was performed by treatment of FTO slides closed in a small container with vapors of MTMOS (trimethoxymethylsilane) and triethylamine in argon atmosphere for about 10 min at room temperature. Spin-coating was performed as follows: MTMOS was mixed with 5 mM HCl in a volume ratio of 5:2, stirred for 2 min, and sonicated for 10 min. Next, the MTMOS–HCl solution was mixed with phosphate buffer (25 mM, pH 8.0) in a volume ratio of 1:1. Subsequently, the solution was spin-coated on previously cleaned glass at 3000 rpm for 30 s. Finally, the slides were dried overnight at room temperature. CVD approach yields relatively thinner and, thus, less hydrophobic layer than spin-coating. The degree of surface hydrophobicity was assessed by measuring the contact angle between a droplet of water and the substrate. These values were ~ 35°, ~ 60°, ~ 45°, and ~ 80° for non-conductive glass surface, bare FTO conductive surface, non-conductive glass surface silanized with CVD method, and FTO glass conductive surface silanized with spin-coating method, respectively. Moreover, the thin layer of the silane obtained by CVD method was not sufficient to significantly increase resistivity of the FTO glass conductive surface. Therefore, this method was used only to increase the hydrophobicity of the non-conducting surface of the FTO glass. The resistivity of the FTO conductive surface silanized by spin-coating was above 20 kΩ/cm in contrast to ~ 30 Ω/cm for bare FTO conductive surface.

For most of the transient absorption experiments with non-conductive surface, we decided to use the system with the conductive surface of FTO glass silanized by spin-coating method (Fig. 1b) in order to maintain the same conditions during the PSI immobilization step (Fig. 2a) as for the sample without silane—PSI on bare FTO (Fig. 1a). In particular, it concerns the same electric field and distance between the electrodes (Fig. 2a).

The cell-like sample (Fig. 1d) was fabricated by the addition of the flexible U-shaped spacer (~ 3 mm of thickness) around the PSI immobilized onto the FTO conducting surface and a microscopic slide. A vacuum grease was placed between the FTO–spacer and spacer–coverslip surfaces to preclude electrolyte leakage. The buffer A solution without β-DM, containing P700 oxidizing agent (potassium ferricyanide; see next paragraph), was poured into the cell. The cell was sealed from the top to prevent vaporization of the solution during transient absorption measurements.

### Femtosecond transient absorption measurements

For time-resolved absorption measurements of PSI in solution, both trimeric and monomeric forms were diluted in buffer A to OD_680 nm, 1 cm_ = 0.4 and placed in the 2-mm quartz cuvette. The RCs in the solution and in the cell system were kept in the closed state of RCs (P700 in the oxidized state, P700^+^) by the addition of 3 mM potassium ferricyanide. The PSI complexes immobilized on substrates were also in closed state, as evidenced by lack of the long-lived P700^+^ signal in transient absorption (see “Results”), characteristic for RC in open state (Savikhin et al. 2000). Control measurements of PSI in solution without oxidizing agent were also performed.

Time-resolved absorption data were collected using Helios transient absorption setup (Ultrafast Systems). Excitation beam was generated by the Ti:Sapphire oscillator (Mai-Tai, Spectra Physics) followed by the regenerative amplifier (Spitfire Ace, Spectra Physics). The amplifier output (800 nm, ~ 100 fs, 1 kHz repetition rate pulses) was split to generate beams: pump (405 nm) in the optical parametric amplifier (Topas Prime) and probe—white-light continuum in the 440–780 nm range using a sapphire crystal. Instrument response function (IRF) was about 200 fs wide.

PSI samples in solution and immobilized on substrates were continuously moved using a mechanical motion controller (Newport) in* X*–*Y* dimensions (directions perpendicular to the incident probe beam), to minimize effects related to excessive irradiation. The phases of cyclic movement in the* X* and* Y* directions were different, so the beams “scanned” the sample in a Lissajous-like pattern. The energy of a single pump pulse was about 15 nJ with a spot size of approximately 200 µm. This excitation level excludes singlet–singlet annihilation effects and is similar to or smaller than that in the previously performed measurements on *Synechocystis* PSI in solution (Savikhin et al. 2000; Melkozernov et al. 2000; Shelaev et al. 2010). Under such conditions, typical absorbance changes in a raw signal were about 1 mOD in maximum, yielding satisfactory signal-to-noise ratio.

The data were acquired in the 2.9-ns time window. Each dataset was measured twice in solution and four times in the case of PSI deposited on substrates (due to relatively higher noise in the latter case) and the datasets were averaged. The results were corrected for background and spectral chirp of white-light continuum using SurfaceXplorer software (Ultrafast Systems). Then global and target analyses were performed using Glotaran software (Snellenburg et al. 2012). Theoretical principles introducing these approaches can be found in van Stokkum et al. (2004).

## Results and discussion

### Transient absorption measurements—kinetics at 690 nm

**Fig. 3:**
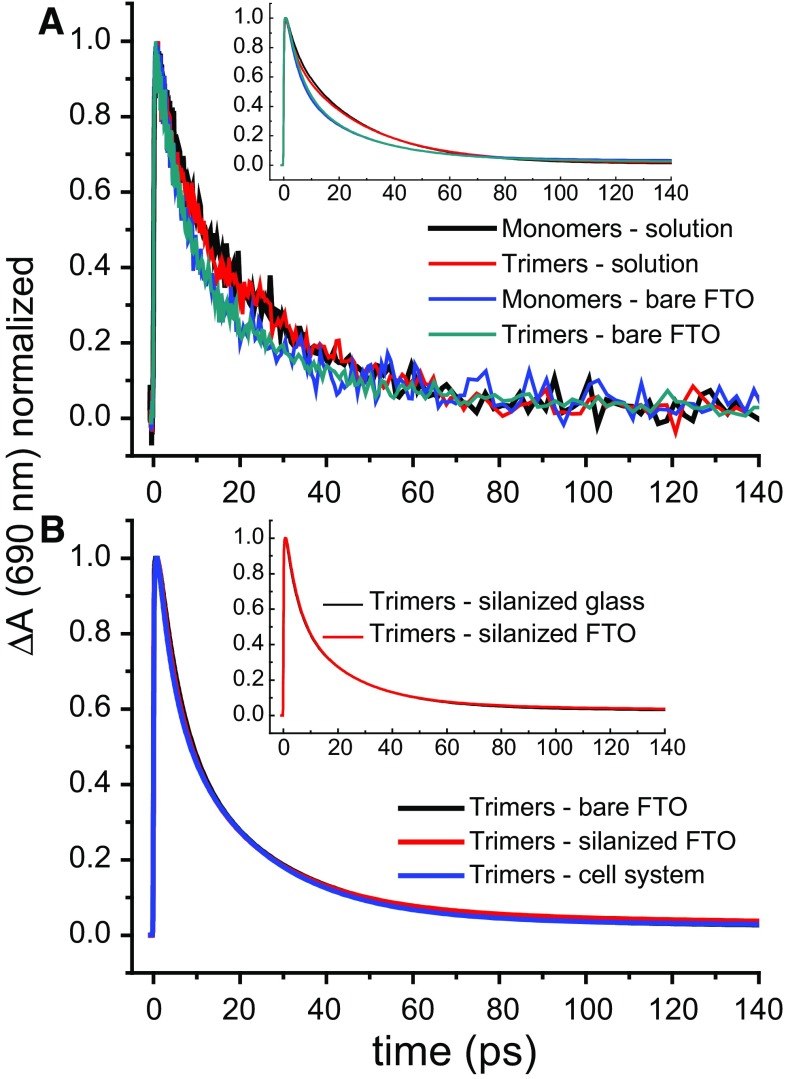
Transient absorption kinetic traces at 690 nm (wavelength of maximal absorbance changes) recorded at an excitation wavelength of 400 nm. The original traces were multiplied by factor (− 1) and normalized to unity. **a** Comparison of raw signals obtained for monomeric and trimeric PSI in solution and immobilized directly onto the conductive surface of FTO glass. Inset: fits to the corresponding data. **b** Data obtained for trimeric PSI immobilized onto substrates in three different systems. Inset: comparison of the decay kinetic for trimeric PSI attached to two different silanized surfaces

Figure 3a presents transient absorption kinetics at 690 nm of monomeric and trimeric PSI in solution and immobilized onto the conductive layer of FTO glass (Fig. 1a). Acceleration of the overall excitation decay in the immobilized PSI versus PSI in solution is well noticeable and consistent with previous, time-resolved fluorescence results (Szewczyk et al. 2017). On the other hand, there are no major differences in kinetic traces between monomeric and trimeric forms, in respective systems. In the following, we focus on trimeric PSI. Similar results for monomeric PSI obtained under identical experimental conditions are shown in supplementary information. Figure 3b presents kinetic traces of the trimeric PSI immobilized onto different substrates/in different systems: PSI deposited and dried on bare FTO (conditions with possible electron injection from PSI to FTO), PSI deposited and dried on silanized FTO (conditions with blocked electron injection by insulating layer of silane), and PSI deposited on bare FTO and being in contact with aqueous buffer. One can see that the traces are almost identical, except for the longest component, the lifetime and amplitude of which slightly vary for different samples (see below). A common feature of these three systems is the formation of a film of crowded PSI complexes. On the other hand, they differ either in electrical contact between PSI and FTO or in degree of hydration. Thus, we hypothesize that the major factor responsible for the acceleration of excitation decay in immobilized PSI complexes is their crowding on the substrate, and not drying or electron injection into the substrate. As shown in the inset of Fig. 3b, the experiments with PSI on silanized FTO and on silanized glass brought also almost identical results. This observation is interpreted in terms of the lack of electron transfer from PSI to the FTO conductive layer through the insulating silane layer by hypothetical tunneling effects or by the silane layer discontinuities.

### Transient absorption measurements—PSI in solution—global analysis

**Fig. 4:**
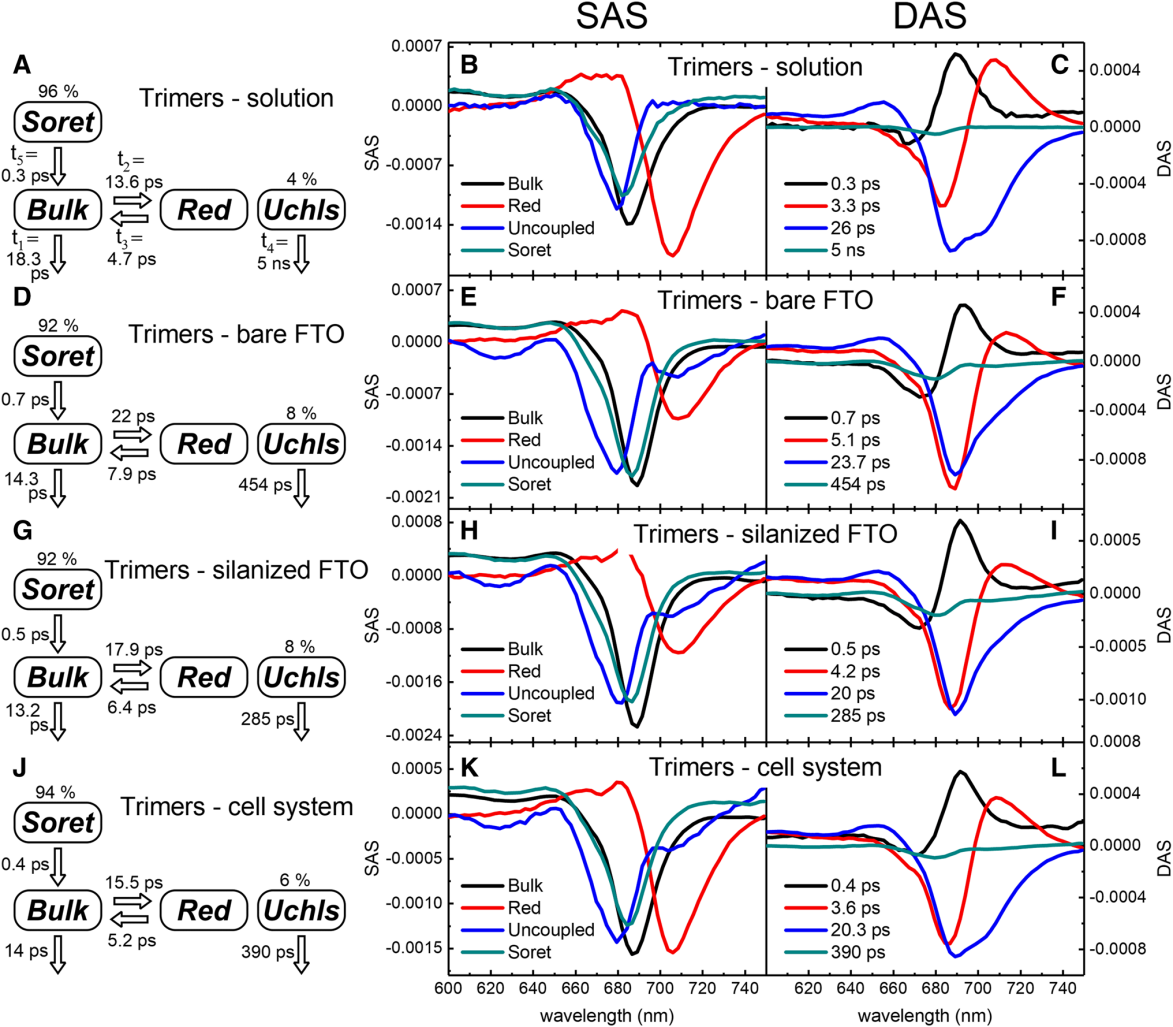
Time-resolved absorption results for the trimeric PSI in solution and immobilized in different systems. The first column (**a, d, g, j**) presents the model underlying target analysis, estimated molecular lifetimes, and initial distribution of the excitation between well-coupled and uncoupled Chls; the middle column (**b, e, h, k**) presents species-associated spectra (SAS) resulting from the target analysis; the third column (**c, f, i, l**) presents the results of global analysis (decay-associated spectra, DAS)

**Fig. 5:**
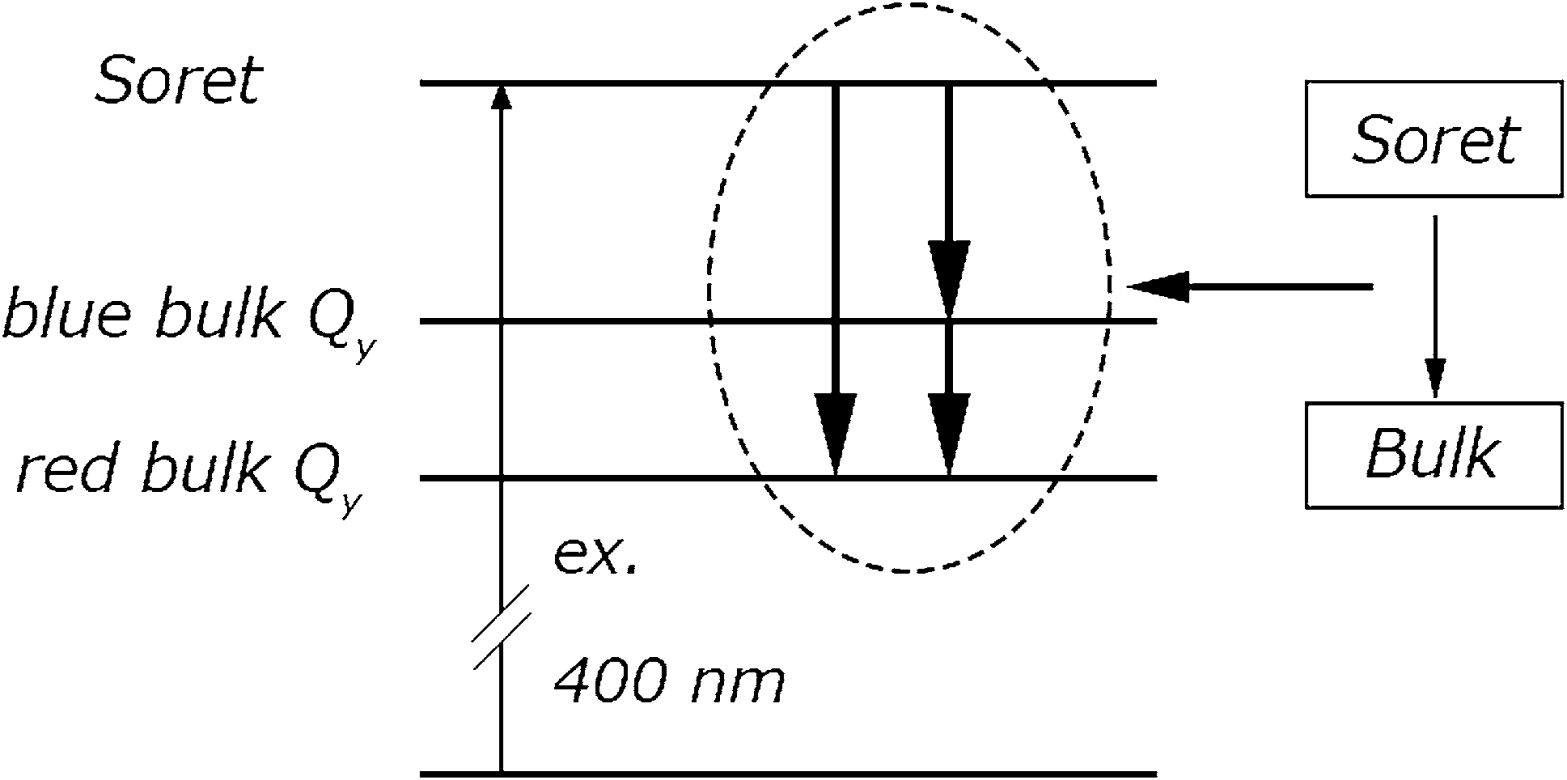
Graphical explanation of the mixed character of the fastest, subpicosecond kinetic DAS and SAS components shown in Fig. 4 as “Soret → Bulk” transition. Within the specified subpicosecond lifetimes in Fig. 4, both relaxation from Soret to Q_y_ states of bulk Chls and excitation transfer between different subpopulations of bulk Chls being in Q_y_ states occur

Figure 4c shows the results of the global analysis performed for trimeric PSI in solution. The dynamics of the PSI complex can be described by three major decay-associated spectral (DAS) components of 0.3, 3.3, and 26 ps. The non-conservative shape of the fastest component with a smaller negative band at about 670 nm and a larger positive band at 690 nm indicates two different processes occurring on the same time scale: (1) relaxation from the Soret to Q_y_ band of Chls (causing the appearance of stimulated emission in the Q_y_ region—manifested as the positive band) and (2) excitation equilibration within the bulk antenna (energy transfer from “blue bulk Q_y_” to “red bulk Q_y_” Chl states, seen as the negative band at ~ 670 nm and a small contribution to the positive band at ~ 690 nm) (Fig. 5). The second component (3.3 ps) was also characterized with two bands, the negative one at about 685 nm and the positive one at about 710 nm. The existence of both positive and negative bands, with similar amplitudes (and integrated areas), indicates energy transfer, which was assigned to equilibration of bulk red Chls. The third component (26 ps) with one negative band at about 690 nm and a bump at about 705 nm was ascribed to photochemical quenching in the RC of the excitation equilibrated over bulk (690 nm) and red (705 nm) Chls. The additional, forth component with a small amplitude was assigned to uncoupled or loosely attached chlorophylls (Uchls), because of their slow excitation decay and blue-shifted spectrum. Similar DAS shapes and lifetimes for the closed RC were reported previously (Savikhin et al. 2000). The slight differences in the spectra reported in our study and those by Savikhin et al. may come from the model used (four- vs. five-component fit), different excitation wavelengths, and different ways of controlling the RC state (chemical vs. strong illumination).

### Transient absorption measurements—PSI in solution—target analysis

In order to obtain more physical description of the excitation energy dynamics in PSI in solution and next in immobilized PSI, the target analysis was performed. Within the given signal-to-noise ratio, the four-compartment model was sufficient to characterize basic transitions for all the systems under study. This model contained the following compartments: “Soret”, “Bulk”, “Red”, and uncoupled (“Uchls”; Fig. 4a). It is essentially the same model as was used previously for analysis of time-resolved fluorescence data (Szewczyk et al. 2017) but with an extra “Soret” compartment which was possible to identify due to better temporal resolution of the transient absorption experiment. As a result, molecular lifetimes of each transition (reciprocals of molecular rate constants; Fig. 4a; Table 1) and spectral distributions of the states within each of the compartments (species-associated spectra (SAS)—Fig. 4b) were obtained. Excitation quenching by closed RC occurs with lifetime* t*_1_ = 18.3 ps (Fig. 4a). Excitation energy transfer from bulk to red Chls with lifetime* t*_2_ = 13.6 ps is coupled with backward transfer which is nearly three times faster than forward reaction:* t*_3_ = 4.7 ps. The SAS band minima for bulk and red Chls are at 685.5 and 706 nm, respectively (Fig. 4b). The negative band of the “Red” SAS is of bigger amplitude than that of the “Bulk” SAS. Similar effect was observed also for monomers in solution (see Fig. S1 in Supplementary information). The blue-shifted spectrum with a maximum at about 680 nm and lifetime* t*_4_ = 5 ns was assigned to uncoupled Chls. The last molecular lifetime,* t*_5_ = 0.3 ps, was ascribed to “Soret” → “Bulk” transition as discussed above (Fig. 5).

**Table 1:** Parameters estimated from transient absorption measurements of trimeric PSI complexes

Sample	Average lifetime at 690 nm *t*_*av*_ (ps)	SAS band minimum wavelength (nm)		*Δλ* (nm) *δ* = ± 0.5 nm	*ΔH* ^*0*^ (meV) *δ* = ± 2.6 meV	*t*_*1*_ (ps)	*t*_*2*_ (ps)	*t*_*3*_ (ps)	*t*_*4*_ (ps)	*t*_*5*_ (ps)	*ΔG* ^*0*^ (meV) *δ* = ± 1 meV	*N*_*r*_^*eff*^
		Bulk *λ*_*b*_	Red *λ*_*r*_									
Trimers solution	20	685.5	706	20.5	53	18.3	13.6	4.7	5000	0.3	− 27	4.3 ± 0.7 (4.2)* (3.9)^#^
Trimers bare FTO	14	689	710	21	53	14.3	22	7.9	454	0.7	− 26	4.3 ± 0.7 (3.9)
Trimers silanized FTO	13	688.5	709	20.5	52	13.2	17.9	6.4	285	0.5	− 26	4.5 ± 0.8
Trimers cell system	13	686.5	706	19.5	50	14	15.5	5.2	390	0.4	− 28	4.6 ± 0.7

Average transient absorption decay lifetime, *t*_av_, was calculated from the equation: *t*_av_ = (*t*_2_ *A*_2_ + *t*_3_ *A*_3_ *)*/*(A*_*2*_ + *A*_3_ *)*, where *t*_i_ are the lifetimes and *A*_*i*_ are the amplitudes (at 690 nm) of the two DAS components (Fig. 4). Bands’ minima were read out from the respective SAS (Fig. 4) and molecular lifetimes, *t*_i_, presented in Fig. 4a were rewritten from Fig. 4. Δ*λ* is the difference between wavelengths of the minima of red and bulk Chls’ SAS. Enthalpy difference (Δ*H* ^*0*^), free energy difference (Δ*G* ^0^), and effective number of red chlorophylls (*N*_r_^eff^) were calculated according to Eqs. S1–S4 (or Fig. S3). In the last column, values in the brackets are the numbers of red chlorophylls reported previously on the basis of time-resolved fluorescence (Szewczyk et al. 2017; indexes “*” and “#” stand for trimeric PSI with open and closed RCs, respectively). The uncertainty of molecular lifetimes necessary to estimate* δ*Δ*G* ^0^ was taken as ± 0.5 ps

### Transient absorption measurements—PSI immobilized on substrates—global analysis

Figure 4f, i, l shows the results of the global analysis obtained for trimeric PSI immobilized in three different systems—onto conductive surface of FTO glass, onto conductive surface of FTO glass covered with insulating silane layer, and onto conductive surface of FTO glass in the cell system, respectively (compare to Fig. 1a, b, d). After PSI immobilization, the three-exponential character of the excitation dynamics is well preserved in all cases. The overall dynamics in all these systems is similar, in line with the results shown in Fig. 3b, and can be described by three major DAS components of 0.4–0.7, 3.6–5.1, and 20–23.7 ps. The most noticeable effects of immobilization are changes in relative amplitudes between the second and the third component (~ 3–5 and ~ 20–24 ps, respectively; compare Fig. 2c with Fig. 2f, i, l). In general, greater contribution of the faster phase over the slower one together with acceleration of the third component after immobilization implies the acceleration of the overall excitation energy decay. Although this effect is partly compensated by increasing lifetime of the second component after immobilization (from 3.3 ps in solution to up to 5.1 ps on bare FTO), direct comparison of the kinetics (Fig. 3) and average lifetimes (Table 1,* t*_av_) demonstrates the overall acceleration effect. The same effect was observed for PSI monomers (Fig. S1 and Table S1 in supplementary information). The non-conservative shape of the second DAS component (3.6–5.1 ps) in the case of immobilized PSI (Fig. 4f, i, l) suggests mixing of two processes: (1) energy transfer assigned to equilibration of bulk red Chls, the same as that for PSI in solution (Fig. 4c) and (2) photochemical quenching of the excitation in the closed RC, absent in solution on this time scale.

The fourth, slowest component is for each PSI-substrate sample characterized by much shorter lifetime (~ 300–500 ps) than in solution (5 ns). These results are in line with those obtained previously with time-resolved fluorescence (Szewczyk et al. 2017) and are discussed below.

### Transient absorption measurements—PSI immobilized on substrates—target analysis

After immobilization of trimeric PSI onto substrates, few main changes relative to PSI in solution can be observed in target analysis results (Fig. 4). The first difference is shortening of the lifetime* t*_1_—from 18.3 ps in solution to 13–14 ps in immobilized PSI. The second one is the weaker coupling between bulk and red Chls reflected by increased values of* t*_2_ and* t*_3_ lifetimes: from 13.6/4.7 ps for PSI in solution to 22/7.9 ps, 17.9 /6.4 ps, and 15.5/5.2 ps for PSI on bare FTO, silanized FTO, and in the cell system, respectively. This effect is quite large for PSI-bare FTO sample, intermediate for PSI-silanized FTO, and weak for PSI in the cell system. The third effect is related to the positions of “Bulk” and “Red” SAS band maxima after PSI immobilization. In solution, the respective bands are at 685.5 and 706 nm. They are red-shifted to 689 and 710 nm for bare FTO and similarly to 688.5 and 709 nm for silanized FTO, respectively. Oppositely, the positions of SAS band maxima for the PSI in the cell system almost did not change in comparison to solution—they are at 686.5 and 706 nm. Also, the differences in amplitudes of SAS are firmly visible. Under “dry” conditions (bare and silanized FTO), the “Red” SAS shows strongly reduced amplitude (compare Fig. 4e, h–b). In immobilized PSI complexes in the cell system (“wet” conditions), the “Red” SAS is also of reduced amplitude albeit to a lower extent (compare Fig. 4k–b). This effect may indicate that the oscillator strength of the red Chls decreases if PSI is immobilized and densely packed. Similar effect was observed in the case of monomeric PSI (Fig. S1) and previously in aggregated (densely packed) LHCII particles in solution (Gruszecki et al. 2006).

Immobilization of PSI in all systems causes similar changes in the lifetime and shape of the uncoupled Chls’ SAS (Fig. 4e, h, k and Fig. S2C). The lifetime is shortened by one order of magnitude as noticed above (global analysis). Apart from main blue-shifted band with maximum at about 680 nm (present also in PSI in solution; Fig. 4b) being a fingerprint of unconnected Chls (Melkozernov et al. 2000), an additional small band at about 705–710 nm can be distinguished. This observation suggests that after immobilization some of the uncoupled Chls undergo transition from “blue” to “red” form. All the described modifications of excitation dynamics induced by immobilization of PSI on the solid substrate are very much consistent with previously published data obtained using time-resolved fluorescence method (Szewczyk et al. 2017).

To sum up, immobilization of PSI causes similar effects in all the systems under study although in the case of the cell system some of the features of SAS (Fig. 4 and Fig. S1) and also DAS (Fig. 4) are intermediate between those for PSI in solution and under the “dry” conditions (bare and silanized FTO).

### Estimation of the effective numbers of red Chls

#### Trimeric PSI

As shown previously (Szewczyk et al. 2017) and in the supplementary information, results of target analysis (molecular lifetimes and spectral positions of SAS; Fig. S3) may be used to estimate energetic parameters of bulk and red Chls (standard enthalpy difference, Δ*H* ^0^, and standard free energy difference, Δ*G* ^0^, between bulk and red Chls), and from those, effective number of bulk and red Chl states may be extracted. The results of these calculations as well as input data taken from the target analysis are shown in Table 1 for PSI trimers and in Table S1 for PSI monomers. The estimated effective number of red Chls per monomer in PSI trimers (4.3–4.6, Table 1) is independent of the system (PSI in solution and different systems with immobilized PSI), which is the same as that reported in the previous fluorescence studies (Szewczyk et al. 2017), from which also a similar number of 3.9–4.2 red Chls per monomer in trimeric PSI was estimated. The results shown in Table 1 consistently demonstrate that despite little spectral red-shift of red (and also bulk) Chls (see also Fig. S1) and the reduction of oscillator strength of red Chls (see above and Fig. 4), immobilization of PSI trimers influences neither the energetic parameters nor the numbers of red Chls.

#### Monomeric PSI

In the case of the monomeric PSI, the results of global and target analyses are generally similar to those for trimers (Fig. S1), except for some differences regarding the red Chls. The estimated effective number of red Chls was increased from 3.1 per monomer in solution to 4.3–5.3 for systems with immobilized PSI (Table S1). Similar tendency was observed in fluorescence studies where immobilization caused an increase in the effective number of red Chls from 3–3.4 in solution to 6.3. In both absorption and fluorescence studies, these changes are related to modifications of energetic parameters, in particular standard free energy difference between bulk and red Chls, Δ*G* ^0^, whose absolute value decreases after immobilization. This observation confirms the previous hypothesis, that Chl–Chl interactions being most likely the origin of the extra red Chl states are more susceptible to modifications for PSI monomers than for trimers after immobilization/dense packing on the substrate (Szewczyk et al. 2017). Appearance of additional low-energy Chls, as a result of aggregation, was also observed previously in LHCII particles (Vasil’ev et al. 1997; Gruszecki et al. 2006; Andreeva et al. 2009; see also below).

### Origin of the acceleration of the excitation energy decay in immobilized PSI

We have performed careful comparative analysis of PSI trimers and monomers immobilized onto different surfaces, conductive and non-conductive, dried, or being in contact with aqueous solution. In all cases, immobilization of PSI complexes caused similar acceleration of excitation decay within the protein. This result suggests that the acceleration of the PSI excitation decay is caused neither by electron injection into the substrate nor by drastically changed hydration state of the proteins, but most likely it is due to dense packing of PSI on the substrate. This conclusion is supported by earlier studies on a different pigment–protein photosynthetic complex, LHCII. For that system, accelerated excitation decay as well as the formation of a few red-shifted chlorophyll species as a result of aggregation, caused by low detergent concentration, was reported (Vasil’ev et al. 1997; Gruszecki et al. 2006; Andreeva et al. 2009). Furthermore, it was proposed that the origin of the new electronic low-energy levels is related to exciton coupling of protein-bound photosynthetic pigments (Gruszecki et al. 2006). Similar effects were observed by us: acceleration of the overall decay—for monomeric and trimeric PSI, and formation of additional red Chls—for monomeric PSI. Moreover, apart from the acceleration of overall excitation decay of Chls well coupled to RC occurring on 10–20-ps time scale (see* t*_av_ values in Table 1 and S1), we also observed ~ 10-fold acceleration of excitation decay within a very minor pool of Chls uncoupled to RC: from ~ 5 ns in solution to ~ 500 ps after immobilization on the substrate. For these reasons, we propose that dense packing of PSI on the substrate resembles the “dense packing” of LHCII complexes within the aggregates, although the exact mechanism of the observed acceleration remains to be discovered. The above consideration was performed for hypothetical homogenous packing, although scenario in which different “clusters” of packed proteins are formed cannot be excluded. The parameters retrieved from the analysis would then represent average/mean values. Finally, we conclude that despite the described spectral and dynamic modifications of PSI complexes immobilized in different systems, these proteins remain fully functional in terms of excitation energy transfer.

## Electronic supplementary material

Below is the link to the electronic supplementary material.

Supplementary material 1 (DOCX 722 KB)
