# Supplementary material for: Acceleration of the excitation decay in Photosystem I immobilized on glass surface
Source: Photosynth Res. 2017 Oct 13;136(2):171–81. doi: 10.1007/s11120-017-0454-z (PMC5895687; doi:10.1007/s11120-017-0454-z)
Supplement: Supplementary file 1 — Supplementary material 1 (DOCX 722 KB) [file 11120_2017_454_MOESM1_ESM.docx]

**Supplementary information**

*Estimation of the effective numbers of bulk and red Chl states*

The “effective” numbers are the hypothetical numbers of isoenergetic bulk states (*N_b_^eff^*) and isoenergetic red states (*N_r_^eff^*) which would give the ratio of energy transfer rates from “Bulk” to “Red” (*k_b→r_* = 1/*t*_2_) and from “Red” to ”Bulk” (*k_r→b_* = 1/*t*_3_) Chls, that is observed in the experiment (see target analysis in Fig. 4 and Fig. S1). The basic thermodynamic equation can be applied in order to estimate these “effective” numbers based on the experimental data (Szewczyk et al. 2017):

$\Delta G^{0}=\Delta H^{0}-k_{B}T\ln\frac{N_{b}^{eff}}{N_{r}^{eff}}$. (S1)

where *k_B_* is the Boltzmann constant, *T* is the absolute temperature, *ΔG^0^* and *ΔH^0^* are the standard (Gibbs) free energy difference ($\Delta G^{0}=G_{b}^{0}-G_{r}^{0}$) and standard enthalpy difference
($\Delta H^{0}=H_{b}^{0}-H_{r}^{0}$) between bulk (index *b*) and red (index *r*) states. Standard free energy difference can be expressed by the ratio of *k_r→b_* and *k_b→r_*, in the following way:

$\Delta G^{0}=-k_{B}T\ln\left(\frac{k_{r\to b}}{k_{b\to r}}\right)=-k_{B}T\ln\left(\frac{t_{2}}{t_{3}}\right)$. (S2)

The standard enthalpy *H^0^* corresponds to the electronic energy levels of the Chls’ excited states. Therefore, the standard enthalpy difference between bulk and red states can be estimated based on the maxima of the respective SASes obtained in target analysis (assuming again isoenergeticity of all bulk and all red Chl states):

$\Delta H^{0}=hc\left(\frac{1}{\lambda_{b}}-\frac{1}{\lambda_{r}}\right)$, (S3)

where *λ_b_* and *λ_r_* are the wavelengths for which SASes of “Bulk” and “Red” Chls, respectively, have got their maxima, *h* is the Planck constant, and *c* is the speed of light in vacuum. In our calculations we applied the normalization condition:

$N_{b}^{eff}+N_{r}^{eff}=100$. (S4)

The more detailed reasoning leading to the above equations can be found in (Szewczyk et al. 2017). Graphical presentation of the above considerations is shown on Fig. S3.

Figures


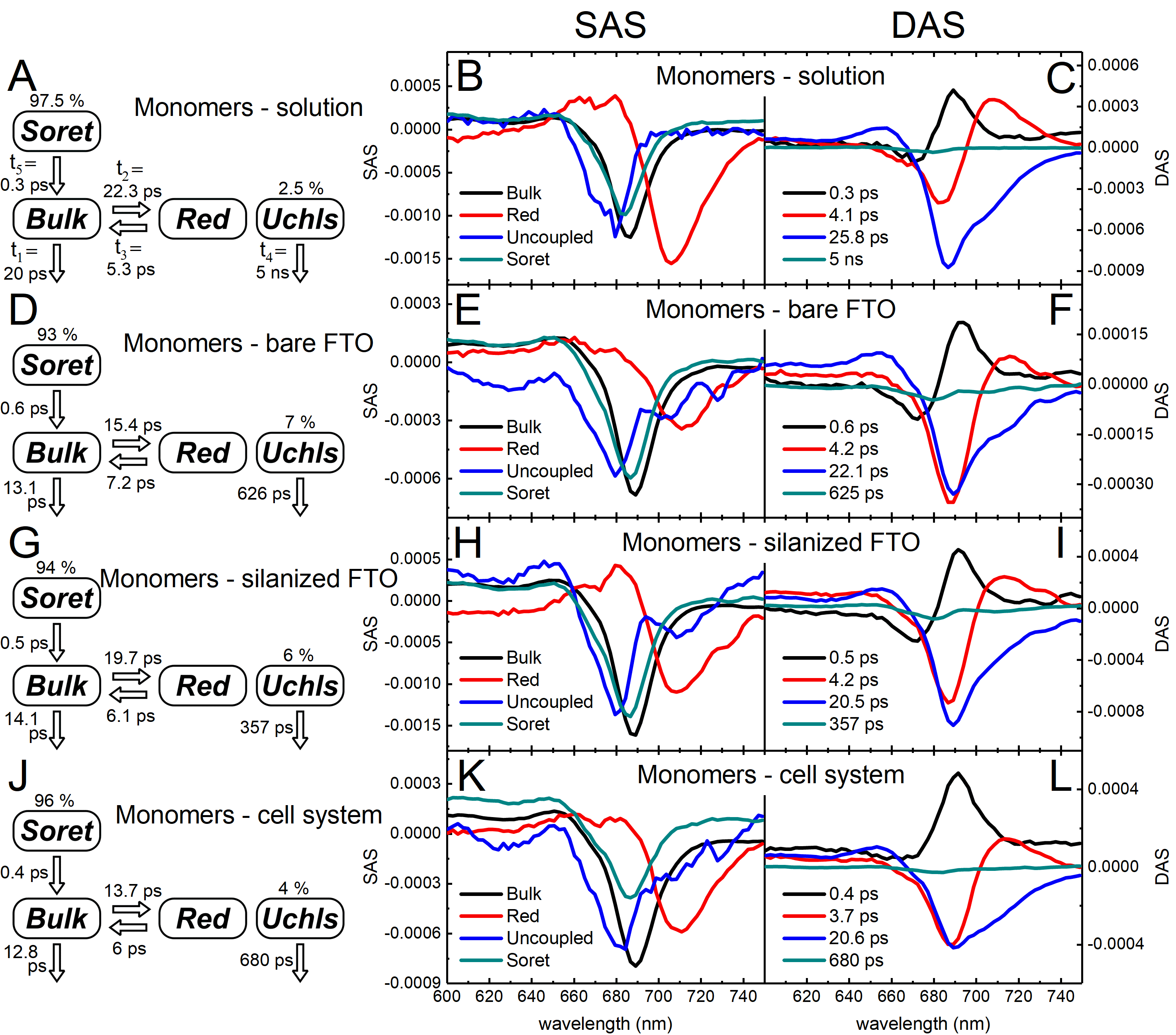


Figure S1. Time-resolved absorption results for the monomeric PSI in solution and immobilized in different systems. The first column (A, D, G, J) presents model underlying target analysis, estimated molecular lifetimes and initial distribution of the excitation between well coupled and uncoupled Chls; the middle column (B, E, H, K) – species associated spectra (SAS) resulting from the target analysis; the third column (C, F, I, L) – results of global analysis (decay associated spectra, DAS).


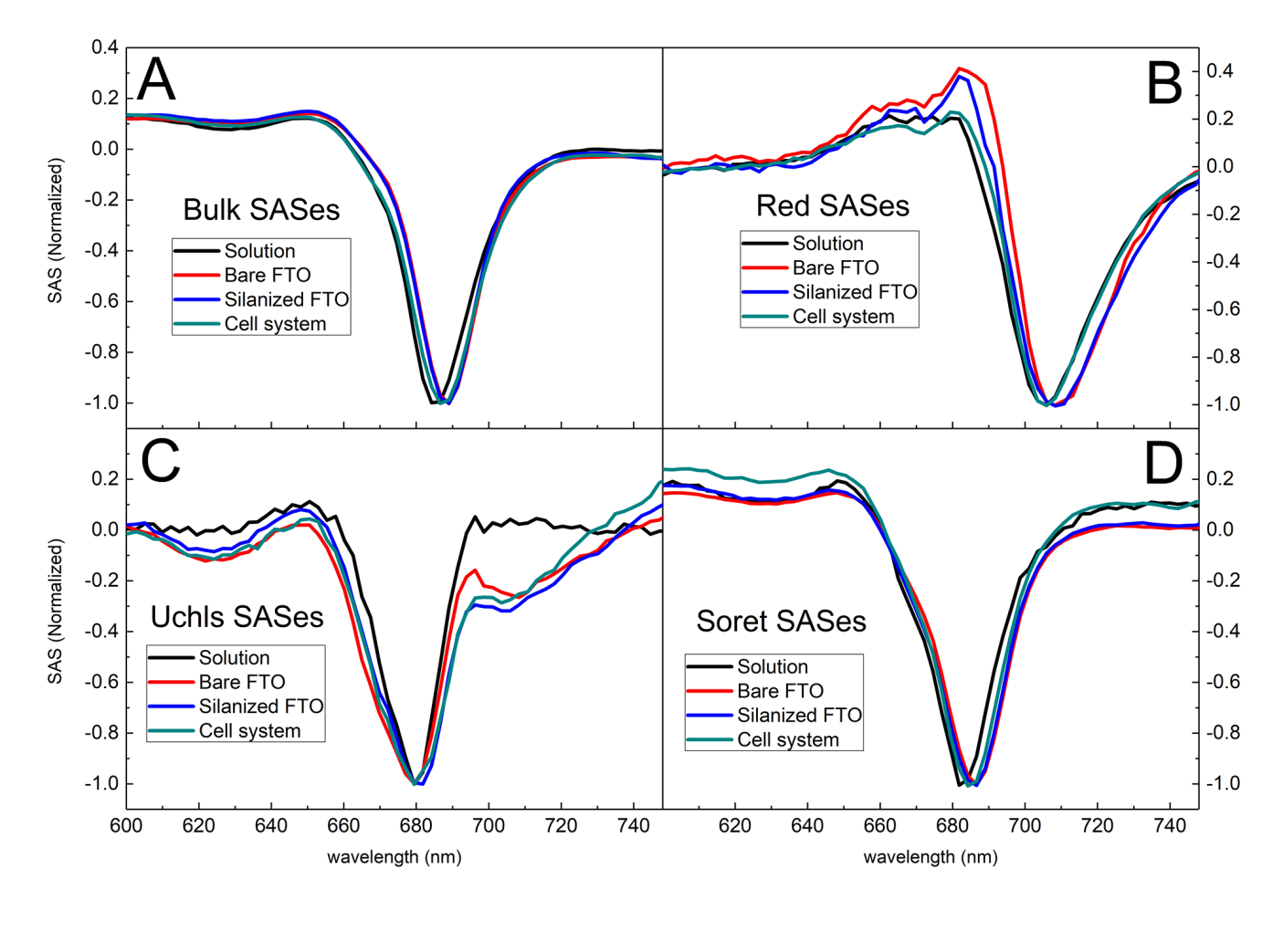


Figure S2. Comparison of the respective SASes obtained for trimeric PSI (redrawn from Fig. 4 and normalized to (-1) in minimum).


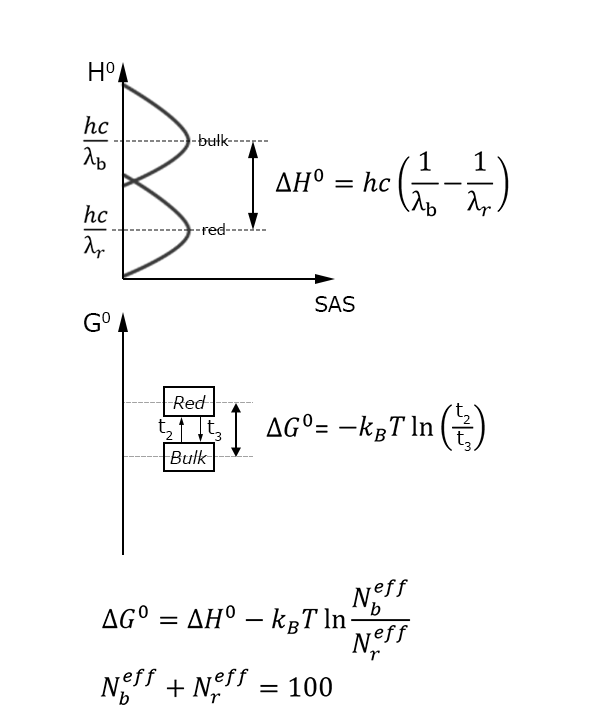


Figure S3. Working energetic model allowing estimation of the effective number of red chlorophylls in all types of samples under study. See text and Szewczyk et al. 2017 for details.

Table S1. Parameters estimated from transient absorption measurements of monomeric PSI complexes.

| Sample | Average lifetime @690 nm  *t_av_* [ps] | SAS band minimum wavelength [nm] | | *Δλ* [nm]  *δ =*  ± 0.5 nm | *ΔH^0^* [meV]  *δ =*  ± 2.6 meV | *t_1_* [ps] | *t_2_* [ps] | *t_3_* [ps] | *t_4_* [ps] | *t_5_* [ps] | *ΔG^0^* [meV]  *δ =*  ± 1 meV | *N_r_^eff^* |
| --- | --- | --- | --- | --- | --- | --- | --- | --- | --- | --- | --- | --- |
|  |  | Bulk  *λ_b_* | Red  *λ_r_* |  |  |  |  |  |  |  |  |  |
| Monomers solution | 21 | 686 | 706 | 20 | 51 | 20 | 22.3 | 5.3 | 5000 | 0.3 | -37 | 3.1±0.5  (3.4)*  (3.0)^#^ |
| Monomers bare FTO | 13 | 688.5 | 710 | 21.5 | 55 | 13.1 | 15.4 | 7.2 | 626 | 0.6 | -20 | 5.3±0.9  (6.3) |
| Monomers silanized FTO | 14 | 689 | 708.5 | 19.5 | 50 | 14.3 | 19.7 | 6.1 | 357 | 0.5 | -30 | 4.3±0.6 |
| Monomers cell system | 13 | 689 | 710 | 21 | 53 | 12.8 | 13.7 | 6 | 390 | 0.4 | -21 | 5.2±0.8 |

Average transient absorption decay lifetime, *t_av_*, was calculated from the equation:
*t_av_* = (*t_2_A_2_+ t_3_A_3_)/( A_2_+ A_3_),* where *t_i_* are lifetimes, and *A_i_* are the amplitudes (at 690 nm) of the two DAS components (Fig. S1). Bands’ minima were read out from the respective SASes (Figs. S1) and molecular lifetimes, *t_i_*, defined in Fig. S1A, were rewritten from Fig. S1. *Δλ* is a difference between the minima of red and bulk Chls SASes. Enthalpy difference (*ΔH^0^*), free energy difference (*ΔG^0^*) and effective number of red chlorophylls (*N_r_^eff^* ) were calculated according to Eqs S1-S4 (or Fig. S3). In the last column, values in the brackets are numbers of red chlorophylls reported previously on the basis of time-resolved fluorescence (Szewczyk et al. 2017; indexes “*” and “#” stand for monomeric PSI with open and closed RC, respectively). The uncertainty of molecular lifetimes necessary to estimate *δΔG^0^* was taken ±0.5 ps.
